# Genome-wide association analyses identify known and novel loci for teat number in Duroc pigs using single-locus and multi-locus models

**DOI:** 10.1186/s12864-020-6742-6

**Published:** 2020-05-07

**Authors:** Zhanwei Zhuang, Rongrong Ding, Longlong Peng, Jie Wu, Yong Ye, Shenping Zhou, Xingwang Wang, Jianping Quan, Enqin Zheng, Gengyuan Cai, Wen Huang, Jie Yang, Zhenfang Wu

**Affiliations:** 1 College of Animal Science and National Engineering Research Center for Breeding Swine Industry, South China Agricultural University, Guangzhou, Guangdong 510642 People’s Republic of China; 2 Department of animal science, Michigan State University, East Lansing, MI USA

**Keywords:** Pigs, Teat number, Multi-locus, GWAS, SNP, VRTN

## Abstract

**Background:**

More teats are necessary for sows to nurse larger litters to provide immunity and nutrient for piglets prior to weaning. Previous studies have reported the strong effect of an insertion mutation in the Vertebrae Development Associated (*VRTN*) gene on *Sus scrofa* chromosome 7 (SSC7) that increased the number of thoracic vertebrae and teat number in pigs. We used genome-wide association studies (GWAS) to map genetic markers and genes associated with teat number in two Duroc pig populations with different genetic backgrounds. A single marker method and several multi-locus methods were utilized. A meta-analysis that combined the effects and *P*-values of 34,681 single nucleotide polymorphisms (SNPs) that were common in the results of single marker GWAS of American and Canadian Duroc pigs was conducted. We also performed association tests between the *VRTN* insertion and teat number in the same populations.

**Results:**

A total of 97 SNPs were found to be associated with teat number. Among these, six, eight and seven SNPs were consistently detected with two, three and four multi-locus methods, respectively. Seven SNPs were concordantly identified between single marker and multi-locus methods. Moreover, 26 SNPs were newly found by multi-locus methods to be associated with teat number. Notably, we detected one consistent quantitative trait locus (QTL) on SSC7 for teat number using single-locus and meta-analysis of GWAS and the top SNP (rs692640845) explained 8.68% phenotypic variance of teat number in the Canadian Duroc pigs. The associations between the *VRTN* insertion and teat number in two Duroc pig populations were substantially weaker. Further analysis revealed that the effect of *VRTN* on teat number may be mediated by its LD with the true causal mutation.

**Conclusions:**

Our study suggested that *VRTN* insertion may not be a strong or the only candidate causal mutation for the QTL on SSC7 for teat number in the analyzed Duroc pig populations. The combination of single-locus and multi-locus GWAS detected additional SNPs that were absent using only one model. The identified SNPs will be useful for the genetic improvement of teat number in pigs by assigning higher weights to associated SNPs in genomic selection.

## Background

Teat number is an important trait for a sow to rear a large number of piglets. A larger litter size in pigs requires sufficient teats for the lactating sow, and the lack thereof can affect the piglets’ weight gain and mortality [1]. Therefore, applying selection on teat number is a useful breeding strategy to improve the reproductive performance of sows in the pig industry [2]. The number of teats has been speculated to be the subject of natural and human-driven artificial selection because it varies substantially between and within breeds [3–5]. Although many broad quantitative trait loci (QTLs) affecting teat number have been identified, the genetic architecture remains elusive [2, 6, 7]. Previous genome-wide association studies (GWAS) in a cross between Landrace and Korean pigs [3], Duroc pigs [4], Erhualian pigs [5], and Large White pigs [7] found that several single nucleotide polymorphisms (SNPs) near or within the Vertebrae Development Associated (*VRTN*) gene on *Sus scrofa* chromosome 7 (SSC7) were associated with teat number. *VRTN* was originally reported as a candidate gene associated with swine vertebral number [8]. The SNP (g.19034A > C) in the promoter and a 291 bp (g.20311_20312ins291) insertion in the first intron of *VRTN* gene could increase the number of thoracic vertebrae in pigs [9, 10]. Recently, Duan et al. [11] showed that *VRTN* mutations influence the thoracic vertebral number, and as a novel transcription factor, the *VRTN* gene is indispensable for the development of thoracic vertebrae in pigs and mice. Furthermore, Yang et al. [12] showed that the 291 bp insertion of *VRTN* has associations with vertebral number, carcass length, and teat number in Chinese indigenous Erhualian pigs, Duroc, Landrace, and Large White pigs. These findings suggested that the 291 bp insertion (g.20311_20312ins291) of the *VRTN* gene seemingly has pleiotropic effects on teat number and several other traits in pigs.

Teat number is a typical polygenic quantitative trait. GWAS using high-density SNPs provides an opportunity to dissect the genetic architecture of such a complex trait by leveraging LD between the causative mutations and common SNP markers [13]. Almost all GWAS for teat number to date employed single-locus models, such as single variant mixed linear model (MLM) [3–5, 7, 14]. Mixed linear model is widely used in association analysis to take account of population structure and genetic relatedness [15–17]. Several recently developed multi-locus models, including the multi-locus random-SNP-effect mixed linear model (mrMLM) [18], fast multiple-locus methods multi-locus random-SNP-effect mixed linear model (FASTmrMLM) [19], fast multi-locus random-SNP-effect efficient mixed model association (FASTmrEMMA) [20], and integrative sure independence screening expectation maximization Bayesian least absolute shrinkage and selection operator model (ISIS EM-BLASSO) [21], were shown to increase statistical power of detecting associations [22, 23].

In this study, we dissected the underlying genetic architecture of teat number in two Duroc pig populations with different genetic backgrounds using single- and multi-locus GWAS in a total 5356 Duroc pigs. Because of the known importance of the g.20311_20312ins291 insertion of the *VRTN* gene, we also specifically tested its association with teat number.

## Results

### SNP genotyping and phenotypic variation

Genotyping was performed using the GeneSeek Porcine 50 K SNP Chip [24]. The quality of genotyping of the 5356 Duroc pigs was examined using PLINK v1.07 [25]. The characteristics of the SNPs in the two populations are summarized in Additional file 1: Table S1, Additional file 2: Table S2, and Additional file 3: Figure S1. These SNPs were roughly proportionally distributed on all 18 chromosomes of pigs, with the longest chromosome having the largest number of SNPs. The average maker densities were approximately 17.81 and 16.49 SNPs per Mb in the American and Canadian Duroc pigs, respectively.

The descriptive statistics of teat number for the 5356 pigs are listed in Table 1. In brief, the average numbers (mean ± standard deviation) of teat number in the American and Canadian Duroc pigs were 10.90 ± 1.16 and 10.92 ± 1.14, respectively. No significant difference in mean teat number was found between the two Duroc pig populations. The coefficient of variation (CV) values for teat number in the American and Canadian Duroc pigs were 10.64 and 10.44%, respectively. Importantly, the SNP-based heritability (*h*^2^ [standard error]) of teat number were 0.19 (0.02) in the American Duroc pigs and 0.34 (0.03) in the Canadian Duroc pigs.

**Table 1:** Summary statistics of teat number in two Duroc pig populations

Population	Trait	N^a^	Mean(±SD)^b^	Min^c^	Max^d^	C.V./%^e^	*h*^2^(±SE)^f^
American Duroc	Teat number	3331	10.90 ± 1.16	9	16	10.64	0.19 ± 0.02
Canadian Duroc	Teat number	2025	10.92 ± 1.14	8	16	10.44	0.34 ± 0.03

^a^Number of animals (N) ^b^ mean (standard deviation) ^c^ minimum (min) ^d^ maximum (max) ^e^ coefficient of variation (C.V.) ^f^ heritability (standard error) value

### Population structure and LD decay

**Fig. 1:**
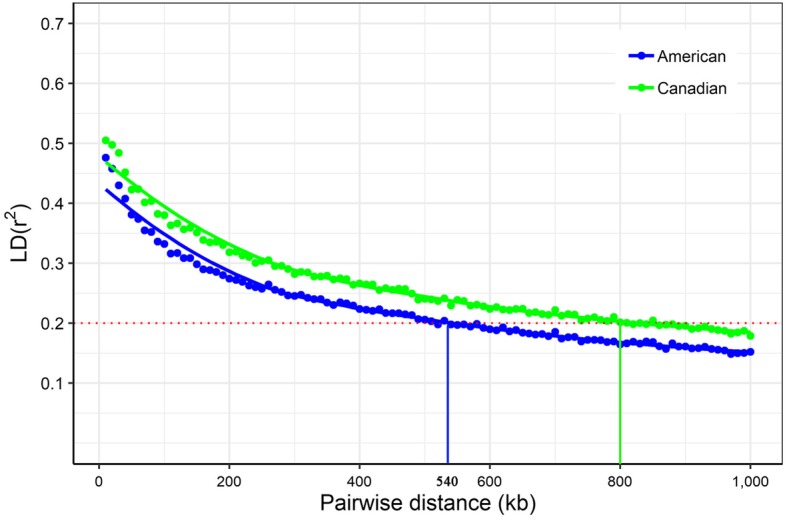
LD decay across the whole genome of the association panel. The red dotted line represents the LD threshold for the association panel (*r*^2^ = 0.2)

In addition to mixed linear model with a covariance among individuals determined by their genotypic relatedness, principal component analysis (PCA) was used to correct for the potential population structure. The first five principal components were fitted as covariates in the association analysis model. In addition, Q-Q plots with genomic inflation factors (λ_gc_) were generated to assess the influence of population structure on the single-locus GWAS (Additional file 4: Figure S2). Systematic inflation of test statistics was not observed for the GWAS of both populations. The average LD decay distances of the American and Canadian Duroc pig populations were approximately 540 kb and 800 kb, respectively, where the *r*^2^ dropped to 0.2 (Fig. 1). Furthermore, pairwise Weir & Cockerham [26] *Fst* value was 0.05 between American and Canadian Duroc pigs, implying little to moderate genetic differentiation [27].

### Single-locus and meta-analysis GWAS for teat number

**Fig. 2:**
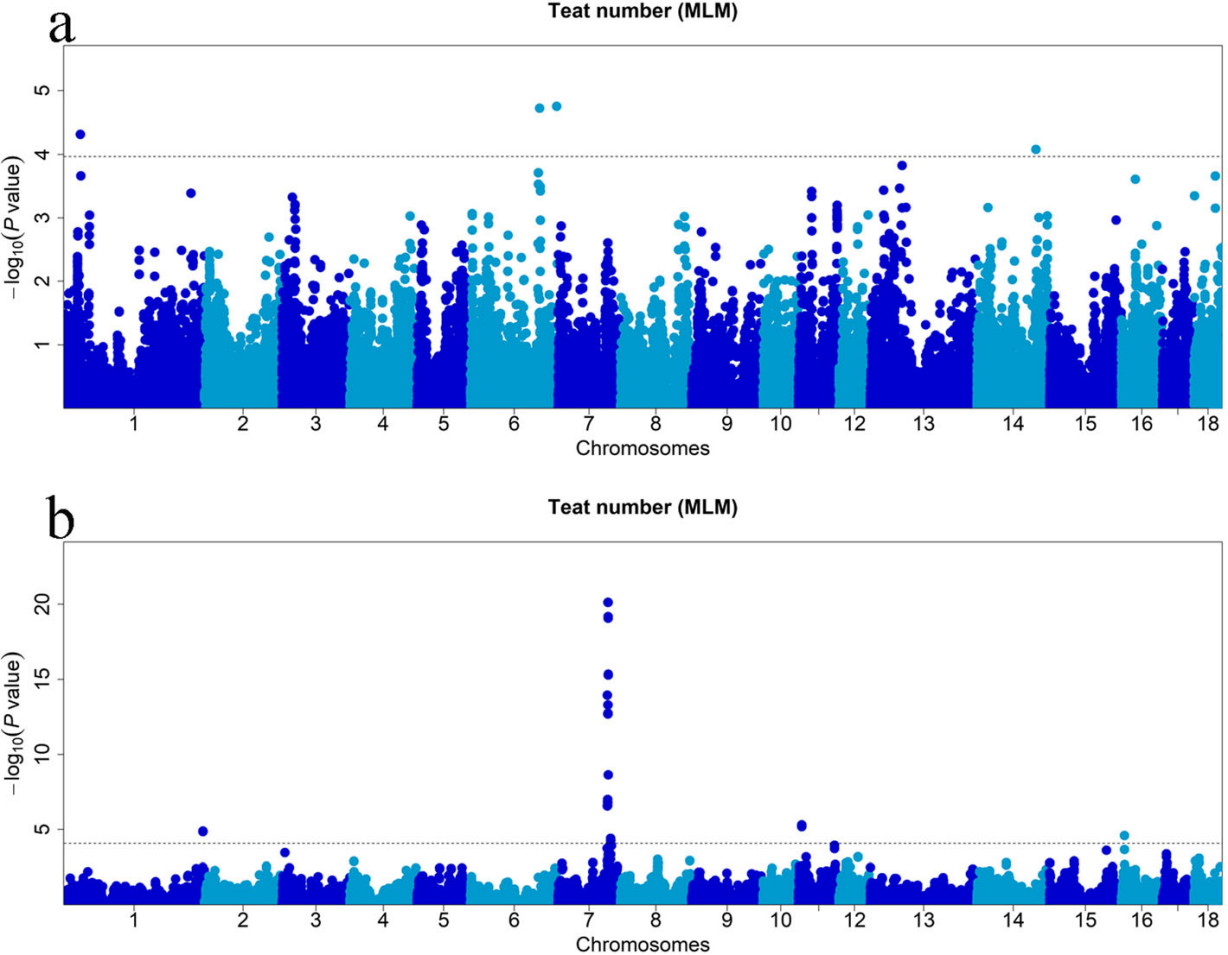
Manhattan plots of the single-locus GWAS for teat number in American (**a**) and Canadian Duroc pigs (**b**). The x-axis represents the chromosomes, and the y-axis represents the -log10(*P-value*). The dashed lines indicate the thresholds for teat number in American (*P* = 1.08E-04) and Canadian (*P* = 8.35E-05) Duroc pigs, respectively

Significant SNPs detected by single-locus GWAS (MLM) for teat number of the American and Canadian Duroc pigs are shown in Table 2 and Table 3, and Fig. 2a and Fig. 2b, respectively. In the American Duroc pigs, two SNPs on SSC6, one SNP on SSC1, and one SNP on SSC14 surpassed the threshold (*P* = 1.08E-04) with an false discovery rate (FDR) controlled at 0.01. Furthermore, the top SNP (rs81391820) accounted for 1.28% of the phenotypic variance. In the Canadian Duroc pigs, 40 SNPs reached the threshold (*P* = 8.35E-05) at an FDR = 0.01, and 35 of which were on SSC7. In addition, two SNPs were located on SSC1, two on SSC11 and one on SSC16. Among these SNPs, rs692640845 on SSC7 explained the most phenotypic variance at 8.68%.

**Table 2:** Significant SNPs associated with teat number in American Duroc pigs

SSC^a^	SNP ID	Position (bp)^b^	MAF	Single-locus GWAS			Multi-locus GWAS			Nearest gene^e^	Distance/bp^f^
				*P-value*	R^2^(%)^c^	Model^d^	LOD	R^2^(%)^c^	Model^d^		
1	rs81296766	26,423,644	0.41				3.23–4.11	0.22–**0.36**	II,**III**,V	*TNFAIP3*	−50,291
1	rs81353367	32,198,014	0.02	4.83E-05	1.94	I				*AKAP7*	−23,051
1	rs81354014	49,632,560	0.41				4.66	0.51	IV	*ADGRB3*	12,781
1	rs80808645	50,037,586	0.3				4.1	1.01	V	*LMBRD1*	59,597
1	rs80855587	166,253,306	0.32				3.18	0.53	V	*ITGA11*	within
1	rs80805477	248,169,556	0.46				3.12–5.68	0.41–**0.68**	**IV**,V	*ZNF462*	within
1	rs81315010	251,816,790	0.14				4	1.39	III	*ENSSSCG00000005457*	within
2	rs81356579	27,110,325	0.5				4.54–5.08	0.56–**0.57**	**II**,III,V	*FBXO3*	−35,449
2	rs330333016	77,481,598	0.11				4.15	0.48	IV	*R3HDM4*	within
2	rs338630193	127,747,767	0.19				3.6	0.73	III	*ZNF608*	−300,480
3	rs81314408	20,394,893	0.11				3.28–4.12	0.80–**1.32**	**II**,III,V	*ENSSSCG00000039406*	−150,340
3	rs81338014	32,426,287	0.05				4.82	2.78	II	*EMP2*	−24,223
4	rs318980859	117,518,657	0.42				3.47	0.32	IV	*VCAM1*	7446
5	rs341491167	10,879,898	0.35				4.38–4.77	0.36–**0.55**	**II**,III,V	*NCF4*	within
5	rs328599079	75,707,707	0.05				3.86	1.36	V	*NELL2*	within
6	rs81395407	35,040,992	0.27				3.12–3.43	0.55–0.80	**II**,III,V	*ZNF423*	within
6	rs333592328	49,265,869	0.27				3.14	0.48	III	*AXL*	within
6	rs81389632	89,786,916	0.05				3.19	2.55	V	*CSMD2*	within
6	rs81391820	134,798,234	0.19	1.87E-05	1.28	I	5.19–7.16	0.62–**3.03**	**II**,III,IV,V	*PTGFR*	21,471
6	rs705289935	168,268,278	0.43	1.74E-05	0.38	I	3.01	0.29	V	*ENSSSCG00000039458*	−13,851
7	rs80964371	92,809,231	0.49				4.08	0.49	V	*DCAF5*	within
9	rs81420227	14,459,452	0.44				3.09	0.17	V	*ENSSSCG00000014896*	−370,357
11	rs81305437	25,782,658	0.15				3.43–4.82	0.94–**1.45**	II,**III**,V	*ENSSSCG00000036698*	within
11	rs80809451	34,924,123	0.04				4.29–5.25	4.23–**4.69**	**II**,III	*ENSSSCG00000040542*	− 248,706
14	rs345307243	24,753,992	0.47				4.57	0.5	IV	*PIWIL1*	within
14	rs80848162	26,467,369	0.49				3.21	0.27	V	*TMEM132C*	within
14	rs80890762	69,437,518	0.21				3.03	0.29	IV	*CTNNA3*	within
14	rs321772507	115,176,455	0.01	8.3E-05	1.11	I				*CFAP43*	2412
14	rs327004523	133,536,115	0.38				3.64	1.07	II	*CHST15*	within
14	rs80794466	137,506,343	0.28				4	0.73	V	*ENSSSCG00000026302*	210,031
15	rs80957887	111,327,896	0.5				3.72	0.41	V	*ENSSSCG00000023264*	within
15	rs333698977	129,904,530	0.27				3.58–4.00	0.33–**0.96**	IV,**V**	*PID1*	− 174,602
16	rs81316660	27,084,056	0.38				3.77–6.24	0.38–**1.90**	**II**,III,IV,V	*GHR*	−42,296
16	rs81461904	69,106,054	0.48				3.22	0.39	V	*MFAP3*	within
17	rs319134655	44,791,974	0.31				3.49	0.44	V	*ENSSSCG00000038990*	within

^a^*Sus scrofa* chromosome ^b^ SNP position in Ensembl ^c^ Proportion of total phenotypic variation explained by each SNP. Bold text indicates the maximum phenotypic variance explained by the multi-locus model ^d^ MLM, mrMLM, FASTmrMLM, FASTmrEMMA and ISIS EM-BLASSO were indicated by I-V, respectively. The bold data represent the model that explained largest phenotypic variance ^e^ Underline indicates that the gene was newly identified as a candidate for teat number ^f^ The SNP located upstream/downstream of the nearest gene

**Table 3:** Significant SNPs associated with teat number in Canadian Duroc pigs

SSC^a^	SNP ID	Position (bp)^b^	MAF	Single-locus GWAS			Multi-locus GWAS			Nearest gene^e^	Distance/bp^f^
				*P-value*	R^2^(%)^c^	Model^d^	LOD	R^2^(%)^c^	Model^d^		
1	rs333890665	271,354,424	0.31	1.28E-05	0.8	I	4.86	1.42	V	*PRRC2B*	within
1	rs321500205	271,382,273	0.45	1.40E-05	0.94	I	4.43–6.09	0.79–**1.47**	II,**III**,IV,V	*ENSSSCG00000022130*	36,815
2	rs81363870	121,140,488	0.49				4.02–6.41	4.77–**7.26**	**II**,III,IV,V	*SEMA6A*	453,157
3	rs344649466	5,715,228	0.35				3.15–3.51	0.78–**1.42**	**II**,III	*NPTX2*	− 5452
5	rs81384813	66,721,316	0.06				3.52	2.54	II	*PRMT8*	within
5	rs81384838	66,734,388	0.1				3.92	1.52	V	*PRMT8*	within
6	rs324552394	164,807,745	0.24				3.58	0.77	V	*EFCAB14*	within
7	rs80864749	7,655,911	0.36				3.24	0.79	III	*ELOVL2*	within
7	rs330783620	9,154,293	0.15				3.18	0.37	IV	*PHACTR1*	−191,156
7	rs80888936	96,128,654	0.35	2.67E-07	3.78	I				*ENSSSCG00000033840*	122,929
7	rs324614194	96,278,617	0.47	1.11E-14	6.07	I				*ENSSSCG00000028159*	−10,186
7	rs331807204	96,632,217	0.36	1.46E-07	3.9	I				*NUMB*	within
7	rs81265875	96,660,861	0.36	1.38E-07	3.92	I				*NUMB*	within
7	rs329434246	96,694,364	0.36	9.91E-08	3.97	I				*NUMB*	within
7	rs81396029	96,727,497	0.36	1.46E-07	3.9	I				*NUMB*	within
7	rs81295281	96,731,838	0.35	2.25E-07	3.79	I				*NUMB*	within
7	rs81396040	96,743,525	0.36	1.50E-07	3.9	I				*NUMB*	within
7	rs81227580	96,786,714	0.35	2.47E-07	3.79	I				*NUMB*	within
7	rs81396043	96,806,775	0.35	2.49E-07	3.77	I				*NUMB*	6800
7	rs342685919	97,048,514	0.44	1.93E-13	5.84	I				*ENSSSCG00000035322*	−708
7	rs80843834	97,109,772	0.35	1.63E-07	3.76	I				*ELMSAN1*	32,738
7	rs80805264	97,126,583	0.35	1.63E-07	3.76	I				*ENSSSCG00000002351*	−47,593
7	rs327357811	97,347,282	0.33	4.97E-14	5.61	I				*BBOF1*	within
7	rs319296259	97,394,296	0.33	1.76E-13	5.5	I				*LIN52*	within
7	rs346287309	97,427,849	0.33	1.99E-13	5.48	I				*LIN52*	within
7	rs692640845	97,568,284	0.48	7.51E-21	8.68	I	36.46	9.31	V	*ABCD4*	within
7	rs1113960993	97,575,068	0.48	7.54E-21	8.68	I				*ABCD4*	within
7	rs330032123	97,584,287	0.48	7.54E-21	8.68	I				*ABCD4*	within
7	VRTN_mutation	97,615,880	0.49	6.81E-20	7.8	I				*VRTN*	within
7	rs343248943	97,617,907	0.48	7.54E-21	8.68	I				*VRTN*	within
7	rs80894106	97,652,632	0.48	8.65E-20	8.34	I				*SYNDIG1L*	− 4101
7	rs81238639	97,946,666	0.46	2.28E-09	5.23	I				*FCF1*	within
7	rs80864705	97,954,258	0.46	2.29E-09	5.23	I				*FCF1*	within
7	rs80929215	97,973,860	0.46	2.29E-09	5.23	I				*YLPM1*	− 2277
7	rs80813473	98,066,911	0.47	4.67E-16	7.73	I				*PROX2*	within
7	rs80836267	98,089,286	0.47	5.07E-16	7.71	I				*DLST*	− 1527
7	rs80865802	102,479,725	0.33	5.99E-05	2.98	I				*ENSSSCG00000021315*	within
7	rs338075156	102,513,443	0.33	3.84E-05	3.01	I				*ENSSSCG00000021315*	within
7	rs80975884	102,552,105	0.33	3.84E-05	3.01	I				*ENSSSCG00000021315*	within
7	rs80822795	102,658,822	0.30	3.96E-05	3.02	I				*ENSSSCG00000021315*	within
7	rs80795811	103,109,678	0.19	5.27E-05	2.82	I				*DIO2*	within
7	NA	103,132,435	0.19	5.27E-05	2.82	I				*DIO2*	within
7	rs80847916	103,151,323	0.19	5.27E-05	2.82	I				*DIO2*	within
7	NA	103,164,950	0.19	5.27E-05	2.82	I				*DIO2*	within
8	rs81401285	72,626,638	0.33				5.75	0.9	IV	*SEPT11*	within
8	rs343488415	73,599,016	0.36				6.52	2.5	II	*FRAS1*	within
8	rs81335362	136,866,026	0.16				3.28	1.1	V	*BMP3*	−12,934
10	rs334392548	16,387,485	0.47				4.03	0.97	II	*SDCCAG8*	within
11	rs80803790	6,291,044	0.26	4.86E-06	1.11	I	3.12–5.39	0.51–**2.39**	**II**,III,IV,V	*MTUS2*	within
11	rs80914601	6,324,834	0.27	6.52E-06	0.93	I				*MTUS2*	within
11	rs343377111	15,473,025	0.36				3.29	1	II	*MRPS31*	801
11	rs80930723	70,370,312	0.29				3.81–4.21	0.62–**1.33**	III,IV,**V**	*FGF14*	within
12	rs81440983	17,695,233	0.33				3.96–4.53	0.84–**1.59**	**II**,III	*WNT3*	within
13	rs335055280	244,235	0.11				3.97–5.35	1.60–**3.59**	**II**,III	*CPNE4*	−123,572
13	rs345752157	198,613,309	0.2				3.42–3.66	0.86–**1.58**	**II**,V	*RUNX1*	within
14	rs345307243	24,753,992	0.39				4.07	0.91	V	*PIWIL1*	within
14	rs81450840	57,407,462	0.24				4.56–6.50	0.77–**2.68**	**II**,III,IV,V	*ENSSSCG00000010164*	−17,318
14	rs80823799	136,408,216	0.18				3.31	0.78	III	*DOCK1*	within
16	rs322985099	6,124,952	0.31	2.53E-05	0.59	I	3.34–4.09	0.48–**1.37**	**II**,III,IV,V	*MYO10*	within
17	rs80843610	8,468,654	0.44				3.00–5.39	0.36–**1.25**	**II**,III,IV,V	*FAT1*	within
18	rs321942793	6,398,431	0.26				3.94–4.24	1.38–**1.99**	**II**,III	*GIMAP2*	within
18	rs81471144	51,455,200	0.41				5.17	1.1	II	*HECW1*	within

^a^*Sus scrofa* chromosome ^b^ SNP position in Ensembl ^c^ Proportion of total phenotypic variation explained by each SNP. Bold text indicates the maximum phenotypic variance explained by the multi-locus model ^d^ MLM, mrMLM, FASTmrMLM, FASTmrEMMA and ISIS EM-BLASSO were indicated by I-V, respectively. The bold data represent the model that explained largest phenotypic variance ^e^ Underline indicates that the gene was newly identified as a candidate for teat number ^f^ The SNP located upstream/downstream of the nearest gene

A meta-analysis that combined the effects and *P*-values of 34,681 SNPs that were common in American and Canadian Duroc pigs was performed. The results of meta-analysis are shown in Additional file 5: Table S3 and Additional file 6: Figure S3. In brief, 28 SNPs were identified as associated with teat number with the threshold of *P* = 1.21E-04. Of these, 27 SNPs were previously highlighted in the single-locus GWAS of the two Duroc pigs and one SNP on SSC18 was newly found to be associated with teat number by meta-analysis of GWAS. Notably, we detected one consistent QTL (rs692640845) on SSC7 for teat number using single-locus and meta-analysis of GWAS.

### Multi-locus GWAS for teat number

**Fig. 3:**
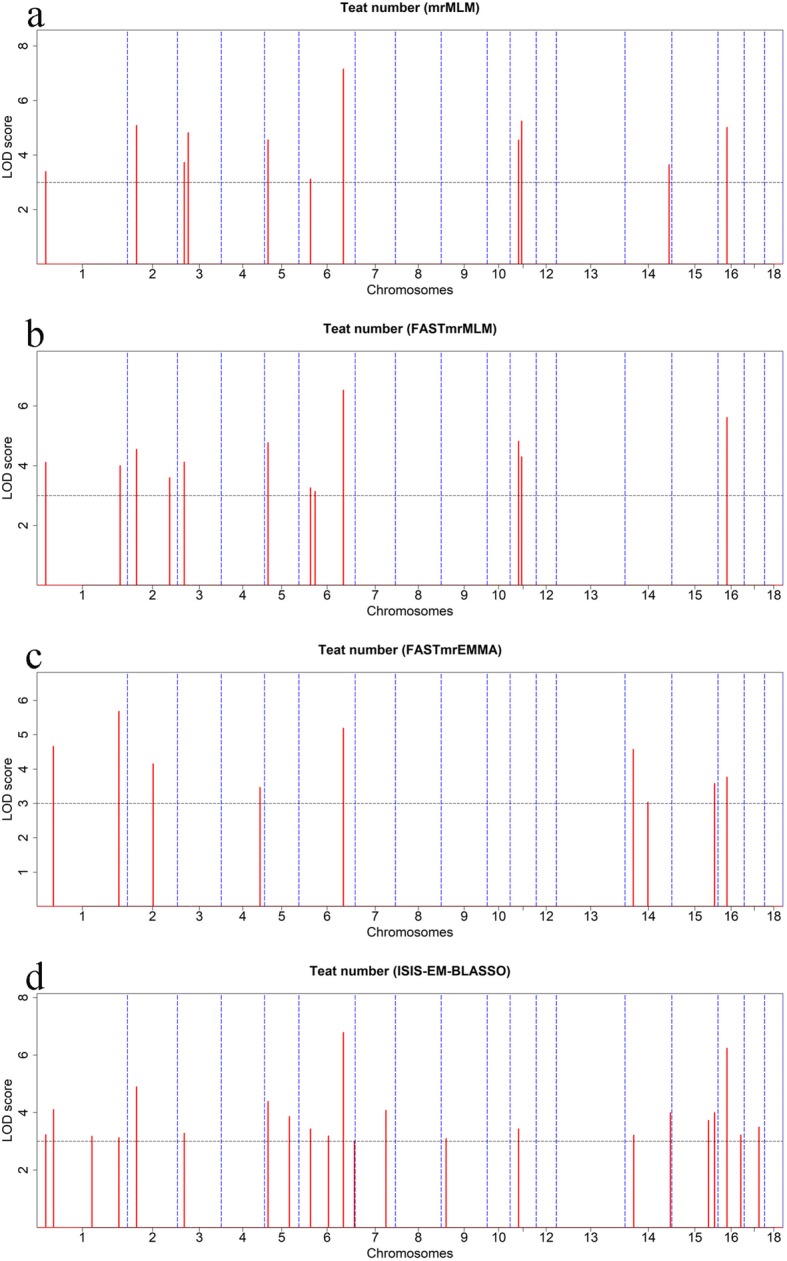
Manhattan plots of the four multi-locus GWAS for teat number in American Duroc pigs. For **a-d**, the Manhattan plots indicate LOD scores for genome-wide SNPs (y-axis) plotted against their respective positions on each chromosome (x-axis), and the horizontal lines indicate the thresholds for significance (LOD score = 3)

**Fig. 4:**
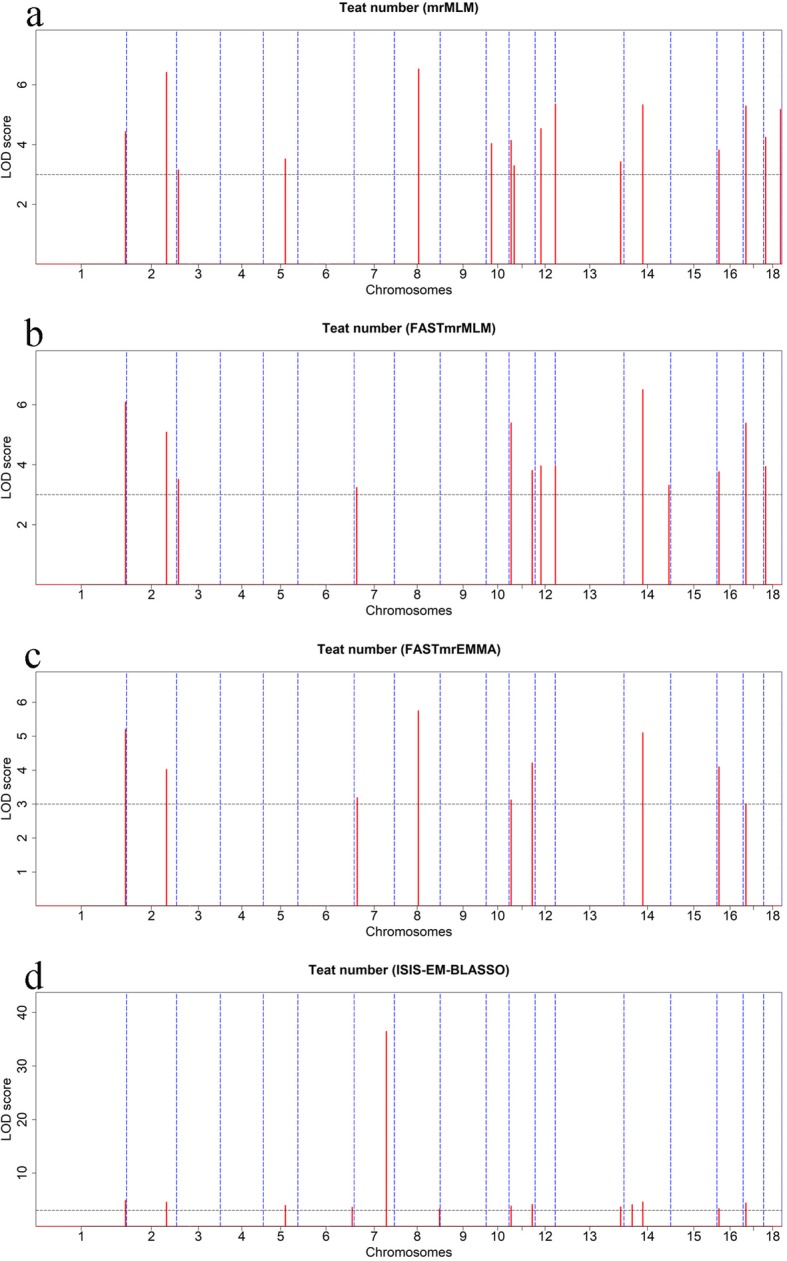
Manhattan plots of the four multi-locus GWAS for teat number in Canadian Duroc pigs. For **a-d**, the Manhattan plots indicate LOD scores for genome-wide SNPs (y-axis) plotted against their respective positions on each chromosome (x-axis), and the horizontal lines indicate the thresholds for significance (LOD score = 3)

**Fig. 5:**
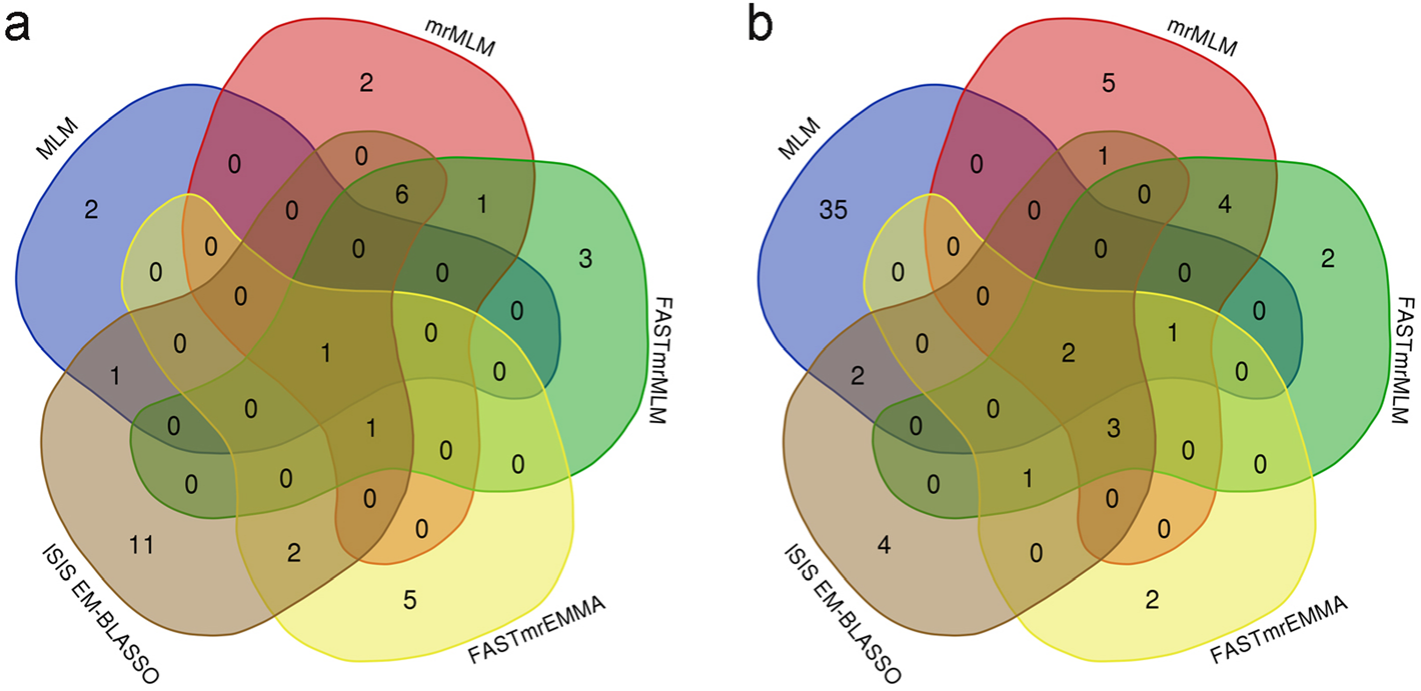
Venn diagrams show the distribution of SNPs from the four multi-locus methods and also highlight the concordance between single marker method and different multi-locus methods. **a**. American Duroc pigs **b**. Canadian Duroc pigs

We next performed multi-locus GWAS using several methods including FASTmrMLM, mrMLM, and FASTmrEMMA, and ISIS EM-BLASSO. In the American Duroc pigs, the four multi-locus GWAS identified 33 teat-number-associated SNPs with at LOD score > 3 (Table 2 and Fig. 3). Among these SNPs, ISIS EM-BLASSO detected the highest number of SNPs (22), followed by FASTmrMLM (12), mrMLM (11), and FASTmrEMMA (9); One, six, and two SNPs were detected by two, three, and four multi-locus models, respectively. Moreover, the two SNPs detected on SSC6 by single-locus MLM were also identified by multi-locus models. In the Canadian Duroc pigs, the four multi-locus GWAS identified a total of 26 teat-number-associated SNPs at LOD score > 3 (Table 3 and Fig. 4). Among these SNPs, mrMLM detected the most SNPs (16), followed by FASTmrMLM (13), ISIS EM-BLASSO (13), and FASTmrEMMA (9); Five, two, and five SNPs were detected by two, three, and four multi-locus models, respectively. The lead SNP rs692640845 was also detected by ISIS EM-BLASSO model with a LOD > 36.46 and explained the 9.31% of phenotypic variance of teat number, implying its strong influence on the teat number trait. Venn diagrams (Fig. 5) show the distribution of SNPs from the four multi-locus methods and also highlight the concordance between single marker method and different multi-locus methods. Briefly, six, eight and seven SNPs were consistently detected with two, three and four multi-locus methods, respectively. Seven SNPs were concordantly identified between single marker and multi-locus methods. Moreover, marker rs345307243 on SSC14, which was found in both populations, was associated with teat number based on FASTmrEMMA and ISIS EM-BLASSO. Notably, the results of multi-locus GWAS for teat number in both Duroc pig populations revealed the 26 SNPs newly associated with teat number that were not previously known (Additional file 7: Table S4).

### Effects of the QTL on SSC7 in two Duroc pig populations

**Fig. 6:**
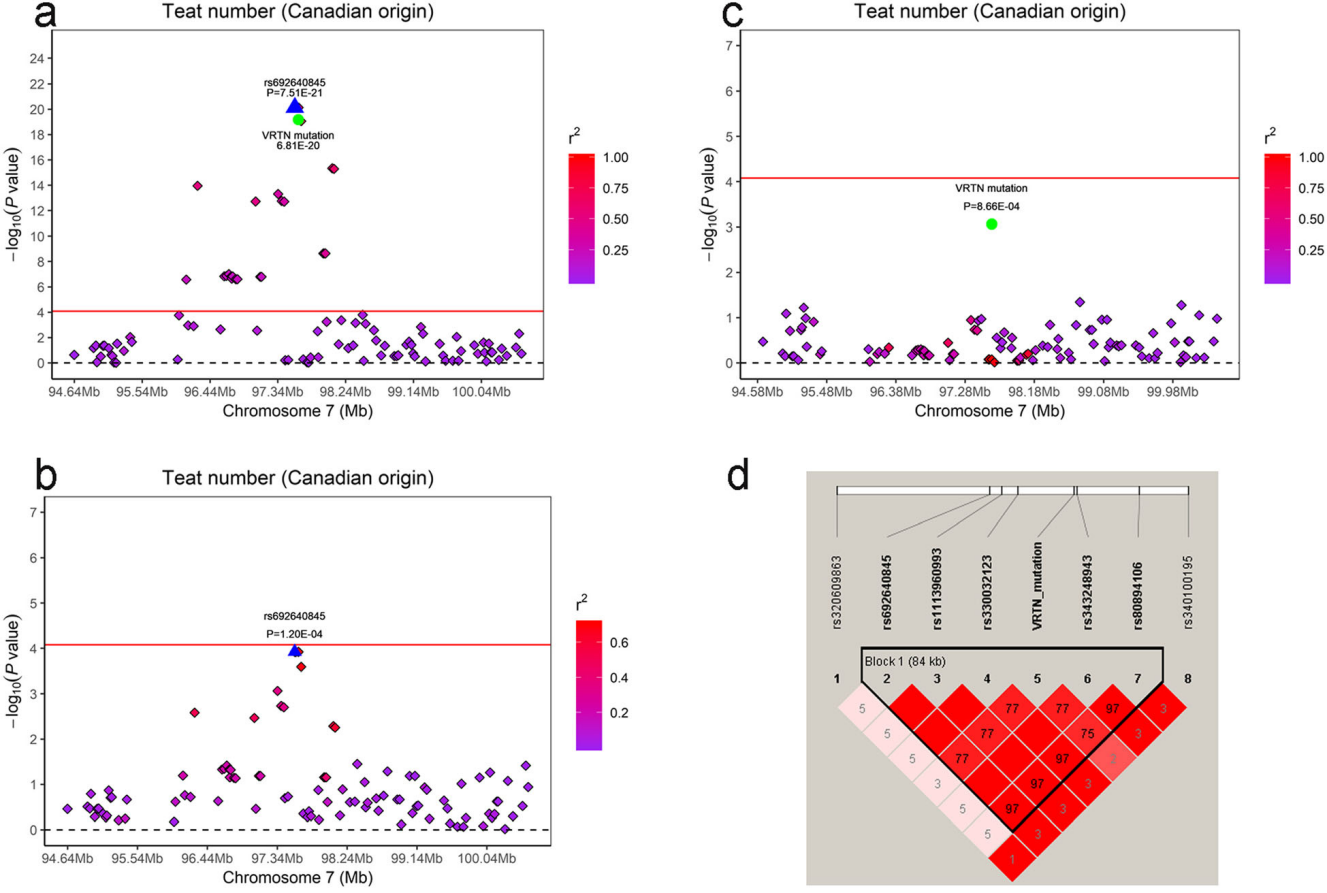
Regional plots of *VRTN* mutation and rs692640845 at 97.48–97.69 Mb on SSC7 for teat number in Canadian Duroc pigs. For **a-c**, the -log10(*P*-values) of SNPs (y-axis) are presented according to their chromosomal position (x-axis). The peak SNP of GWAS is denoted by large blue triangles. The *VRTN* mutation is denoted by green dot. Other SNPs are represented by colored rhombi according to the target SNP with which they were in strongest LD. The plots of **a** and **b** shows the association results for teat number before and after conditional analysis on *VRTN* mutation. The plots of **a** and **c** shows the association results for teat number before and after conditional analysis on rs692640845. **d** represents the 84 kb LD block in the significant region on SSC7 (97.48–97.69 Mb). The complete red boxes with no numbers indicated that SNP pairs have complete linkage disequilibrium

Associations between the *VRTN* mutation and teat number in the two Duroc pig populations were analyzed using single-locus model (MLM), which revealed that no association (*P* = 0.032) between the *VRTN* genotype and teat number in the American Duroc pigs. Although the *VRTN* insertion was strongly associated with teat number in the Canadian Duroc pigs, the effect was weaker than the top SNP (rs692640845) identified in this population (Fig. 6a). To determine whether the signal in this QTL region (96.1–98.2 Mb) was caused by the *VRTN* mutation or the top SNP rs692640845, we conducted conditional analyses by adding the genotypes of these two variants at each locus in the MLM as covariate. As illustrated in Fig. 6b and c, association between the significant SNPs in this QTL region and teat number was greatly diminished in the presence of the *VRTN* mutation or rs692640845 as a covariate. However, a slight signal remained (rs692640845: *P* = 1.20E-04) when the *VRTN* mutation was included in the model but not the rs692640845 SNP. Furthermore, we evaluated the LD pattern of the significant SNPs within the QTL region. Almost all of the significant SNPs in the 84 kb haplotype block were in complete LD except the *VRTN* mutation (Fig. 6d).

We also evaluated the phenotype distribution pattern of the *VRTN* alleles in the two Duroc pig populations (Table 4). Both American and Canadian Duroc pigs were segregating for the *VRTN* mutation. In the Canadian Duroc pigs, the mutant allele (*ins*) had an increasing effect relative to the wild-type allele (*del*) on teat number. The average teat number of the ins/ins pigs was 11.42 ± 1.22, which was 0.88 more than that of del/del individuals with average teat number of 10.54 ± 1.02. The effect was consistent with rs692640845 (AA vs GG: 11.38 ± 1.19 vs 10.50 ± 1.03). In the American Duroc pigs, the average teat number of *ins/ins* and *del/del* pigs was 10.90 ± 1.00 and 10.56 ± 0.79, respectively. These findings suggested that *VRTN* may not be a strong or the only candidate causal gene for teat number in the two pig populations.

**Table 4:** The frequencies and associations of the *VRTN* mutation (g.20311_20312ins291) with teat number in two Duroc pig populations

Population	No. of animals	Genotype frequency ^a^			Allele frequency		Phenotypic value ^b^			R^2^ (%)	*P-value*
		*ins/ins*	*ins/del*	*del/del*	*ins*	*del*	*ins/ins*	*ins/del*	*del/del*		
American Duroc	1496	0.66 (988)	0.31 (460)	0.03 (48)	0.82	0.18	10.90 ± 1.00	10.77 ± 0.97	10.56 ± 0.79	0.50	0.032
Canadian Duroc	2025	0.24 (479)	0.51 (1034)	0.25 (512)	0.49	0.51	11.42 ± 1.22	10.86 ± 1.06	10.54 ± 1.02	7.80	6.81E-20

^a^Frequency of each genotype and the mutant allele (ins) associated with increased teat number at the *VRTN* mutation site are shown in this table. The number of pigs within each genotype is given in parentheses ^b^ Phenotypic values are shown in mean ± standard deviation

### Candidate genes search and functional annotation

Among the identified 97 SNPs, 62 SNPs were located within 44 genes and 35 SNP were not located within any genes but at an interval of 708 bp to 453.16 kb to the nearest genes (Table 2 and Table 3). Considering the genome-wide LD decay distance of the American and Canadian Duroc pigs used in the present study, genomic regions within 540 kb and 800 kb on either side of the 97 SNPs were used to mine candidate genes for teat number, respectively. To understand further the functions of genes implicated by the GWAS, a final set of 426 genes within the LD regions of these SNPs were functionally annotated (Additional file 8: Table S5, Additional file 9: Table S6). Gene set enrichment analysis revealed many terms might be relevant with teat number (Additional file 10: Table S7). Furthermore, the functions of these genes involved in the highlighted terms were identified from GeneCards database and literatures. Five genes in the LD decay range of the QTL on SSC7 including ATP binding cassette subfamily D member 4 (*ABCD4*), YLP motif containing 1 (*YLPM1*), NUMB endocytic adaptor protein (*NUMB*), Prostaglandin Reductase 2 (*PTGR2*), and Apoptosis Resistant E3 Ubiquitin Protein Ligase 1 (*AREL1*) were further highlighted as promising candidates for teat number in pigs.

## Discussion

### Genetic background can affect GWAS

In this study, we carried out GWAS of teat number with a panel of 5356 Duroc pigs using one single-locus model (MLM) and four multi-locus models (mrMLM, FASTmrMLM, FASTmrEMMA and ISIS EM-BLASSO). The combination of single-locus and multi-locus models significantly increased the power of GWAS and detected 97 significant genetic markers. According to the results of GWAS, many candidate genes were annotated using a series of bioinformatics analyses and functional annotations. The findings provide new insight into further deciphering the genetic architecture of teat number in pigs. The experimental animals used in this study consisted of two Duroc pig populations with different genetic backgrounds. Although these animals are of the same breed and are closely related, the genome-wide LD decay distances of American origin and Canadian origin populations were 540 kb and 800 kb, respectively, suggesting different LD patterns. Moreover, heritability estimates of the trait in the two populations were approximately 0.2 and 0.42 [1, 28], which were lower than the estimated values in a previous study [14]. The *Fst* estimate of 0.05 between American and Canadian Duroc pigs demonstrates little to moderate genetic differentiation [27]. However, the GWAS results for teat number in American and Canadian Duroc pigs differed substantially. For instance, the frequency of the ins/ins genotype of *VRTN* insertion in American Duroc pigs was 0.66, which was 0.24 in Canadian Duroc pigs. This result suggests that genetic heterogeneity of *VRTN* insertion may exist in different populations. The genetic drift and the exchange of genetic material that presumably occurred in the two populations [27, 29], provided an explanation for the existence of difference in genetic background can have substantial effect on genetic variant mapping.

### Superiority of multi-locus GWAS and new SNPs for teat number

With the development of molecular markers, GWAS have been widely used to understand the genetic basis of complex traits in animals [30]. Most studies focused only on single-locus GWAS models. Nevertheless, multi-locus models have become popular owing to presumably higher power after accounting for multiple QTLs [23]. For instance, Li et al. [23] performed GWAS to dissect the genetic architecture of fiber quality traits in upland cotton (*Gossypium hirsutum* L*.*) using three single-locus models including GLM [31], MLM [31], and CMLM [17] and three multi-locus models including mrMLM [18], FASTmrEMMA [20], and ISIS EM-BLASSO [21]. The six models totally detected 342 significant SNPs and approximately 85% of the SNPs were identified by multi-locus models. In the present study, using single-locus GWAS (MLM), we initially detected only four SNPs and 40 SNPs in the American and Canadian Duroc pigs, respectively. To improve the efficiency of the study, we performed multi-locus GWAS on teat number of the two Duroc pig populations. The combination of single-locus and multi-locus GWAS detected additional SNPs in comparison with that using only one model, i.e., 33 SNPs were associated with teat number by mrMLM, FASTmrMLM, FASTmrEMMA, and ISIS EM-BLASSO, and two SNPs identified by MLM were also highlighted in the American Duroc pigs. In this study, the single-locus method has less power for detecting the SNPs with minor effects and ignores the presence of additional QTLs on quantitative traits. However, multi-locus models consider multiple QTLs in the model and treat them as random effects. This is much closer to the fact that multiple genes controlled the phenotypes and may increase detection effectiveness [32]. In the results of four multi-locus GWAS, the concordant SNPs are limited. ISIS EM-BLASSO demonstrated the higher power of detecting trait-associated SNPs in both two Duroc pigs and was presumably due to the good performance for dimensionality reduction of large data set in the first stage and integrating EM- Bayesian LASSO algorithm to select and estimate effects in the final stage [21]. Among the 97 significant SNPs, 25 were detected by at least two models, implying that they may be more confident candidates. The marker rs692640845 (SSC7: 97568284 bp) detected by ISIS EM-BLASSO model (also detected by single-locus GWAS) with LOD > 36.46 accounted for 9.31% phenotypic variance of teat number. The results also suggested that fine mapping and functional experiments are needed in the region adjacent to rs692640845 to uncover the true causative mutation. Our findings demonstrated that combining single-locus and multi-locus models of GWAS is an effective strategy for deciphering the genetic basis of complex traits in animals. Many QTLs or SNPs related to teat number have been identified using linkage mapping and GWAS in pigs [2, 33–35]. To evaluate whether SNPs associated with the teat number in the present study replicate any previously known QTLs, we compared the significant SNPs from this study with SNPs in the pigQTLdatabase (https://www.animalgenome.org/cgi-bin/QTLdb/SS/index) based on the location of SNPs. The 28 SNPs newly associated with teat number were not found in any previously known QTLs (Additional file 7: Table S4) using single-locus, multi-locus, and meta-analysis methods, and the genes nearest to these SNPs were considered to be promising candidates for teat number in pigs (underlined gene names in Table 2 and Table 3). Based on a large population size (*n* = 5356) and multiple association analysis strategies, 26 newly identified SNPs were found by the four multi-locus methods. These SNPs each explained a small proportion of the phenotypic variance of teat number ranged from 0.17 to 3.59%, suggesting that sample size and multi-locus methods of GWAS may have played an important role in detecting SNPs with small effects on teat number than previous studies that used smaller sample size and single-locus models.

### Consistent QTL for teat number on SSC7 detected by GWAS

The 27 SNPs associated with teat number were located in a consistent QTL hotpot on SSC7 between 96.1 Mb and 98.2 Mb (*Sscrofa* 11.1). Previous studies consistently reported the influence of the *VRTN* gene on teat number in diverse pig breeds [7, 14, 36]. However, they did not directly perform association analyses between *VRTN* mutation and teat number. Here, we examined *VRTN* mutation genotypes in a larger sample size (3521 Duroc pigs) than in a previous study [12] and conducted association analysis between *VRTN* mutation and teat number. Association analysis between *VRTN* mutation and teat number in the present two Duroc pig populations demonstrated that *VRTN* mutation was not the peak site for teat number. The top SNP rs692640845 within *ABCD4* is located 46.4 kb upstream of *VRTN*. Further analysis implied that the effect of *VRTN* on teat number may be induced by the presence of high LD with the true causal mutation in this QTL region. Teat development is subjected to the formation of the mammary gland in the embryo [37, 38]. Mammary glands appear and develop in pairs together with two normally symmetrical lines along the ventrolateral boundary of the embryo and become functional in adults [37, 39]. Recently, *VRTN* has been characterized as a novel DNA-binding transcription factor and is essential for the development of thoracic vertebrae at somitogenesis stage in pigs and mice [11]. Therefore, we speculated that *VRTN* may not be a strong or the only causal gene affecting nipple formation. Laboratory functional experiments are needed to test this assumption. In an attempt to uncover the promising candidate genes of teat number for this consistent QTL, we searched several genes based on the LD decay distance of the SNPs in the QTL region. *ABCD4* is a member of the superfamily of ATP-binding cassette (ABC) transporters and is involved in intracellular processing of vitamin B12 [40]. Vitamin B12 is a cofactor in methionine synthase and the fortification thereof can prevent neural tube defects in humans [38, 41]. The link between neural tube and mammary gland development may be established through Neogenin, which regulated a range of developmental processes [42]. *YLPM1* plays an important role in the reduction of telomerase activity during differentiation of embryonic stem cells [43]. Verardo et al. [6] have previously reported *YLPM1* as a candidate gene for teat number in a commercial pig line. *NUMB* is a protein coding gene and participates in the determination of cell fates during development [44]. Intriguingly there is seemingly a link between *NUMB* and *VRTN* gene under the Notch signal pathway, which plays a role in the differentiation of nerve cells and the development of vertebrates segments [11, 45]. *PTGR2* encodes an enzyme involved in the metabolism of prostaglandins [46]. There is no evidence that *PTGR2* and an adjacent gene, *AREL1*, are involved in the regulation of mammary gland development. However, a previous study reported that many SNPs within or near the *PTGR2* and *AREL1* genes showing the largest contributions to the genomic heritability and prediction accuracy for teat number in a Duroc pig populations [14]. In further analyses, large-scale transcriptome data and proteome data, as well as laboratory functional experiments, are necessary to pinpoint the causal gene(s) for nipple formation and teat number.

## Conclusions

In conclusion, 37 and 52 SNPs were found to be associated with teat number by only using single-locus and multi-locus methods, respectively, combining the results of both Duroc pigs. The integration of single-locus and multi-locus GWAS detected additional SNPs in comparison with that using only one model. Considerable differences in GWAS results were identified in the American (35 SNPs) and Canadian (62 SNPs) Duroc pigs. These findings demonstrated that the potential genetic differentiation of the two Duroc pig populations can have substantial effect on genetic variant mapping. Furthermore, the associations between the *VRTN* insertion and teat number in two Duroc pig populations were substantially weaker. These findings revealed the complexity of the genetic mechanism of teat number, and provide essential insights into future molecular breeding of pigs in the context of genomic selection.

## Methods

### Ethics statement

All animals used in this study met the guidelines for the care and use of experimental animals established by the Ministry of Agriculture of China. The whole study was approved by the ethics committee of South China Agriculture University (SCAU, Guangzhou, China). The experimental animals were not anesthetized or euthanized in order to conduct this study.

### Sample collection and phenotyping

In the current study, experiment animals were raised in two core breeding farms of Wens Foodstuff Group Co., Ltd. (Guangdong, China). In brief, a total of 5356 Duroc pigs comprising 3331 (2154 males and 1177 females) of American origin born between 2013 and 2017, and 2025 (1018 males and 1007 females) of Canadian origin born between 2015 and 2017 were analyzed. All pigs were subjected to the same growth and feeding condition. The left and right teat numbers were collected by counting separately after birth. In this study, teat number was the sum of teat numbers on both sides. Outliers beyond 3 standard deviations were removed prior to association analysis.

### Genotyping and quality control

The genomic DNA of each pig from ear tissue was isolated following the standard phenol/chloroform method, quantified, and diluted to 50 ng/μL [47]. Genotyping was performed using the GeneSeek Porcine 50 K SNP Chip (Neogen, Lincoln, NE, USA) [24]. A total of 50,915 SNPs were genotyped. Quality control (QC) was performed using PLINK v1.07 [25]. Individuals with call rates smaller than 95%, and SNPs with call rates smaller than 99% and minor allele frequency smaller than 0.01 were also removed. SNPs that failed the Hardy-Weinberg equilibrium test (*P* < 10^− 6^) and unmapped or located on the sex chromosomes were also excluded. After QC, a final set of 38,873 SNPs for 3331 American Duroc pigs and 35,933 SNPs for the 2025 of Canadian Duroc pigs were retained for subsequent analyses.

The *VRTN* g.20311_g.20312ins291 (SSC7:97615880) mutation (referred to as the *VRTN* mutation or insertion hereafter) on SSC7 was genotyped using a PCR-based test. In brief, a total of 3521 Duroc pigs including 1496 American Duroc pigs and 2025 Canadian Duroc pigs were genotyped for the *VRTN* mutation. The sequences for the *VRTN*-FP and *VRTN-RP* primers were GGCAGGGAAGGTGTTTGTTA and GACTGGCCTCTGTCCCTTG, respectively. PCR reaction was described in our previous study [12]. In brief, amplification was carried out in a 25-μL reaction mixture consisting of 0.8 μL DNA sample, 1.5 μL MgCl2, 2.5 μL Buffer, 2.5 U Taq and 1.0 μL forward and reverse primers under the thermocycle condition of 95 °C for 5 min, 35 × (95 °C for 30 s, annealing temperature of 60 °C for 30 s and 72 °C for 30 s) and 72 °C for 10 min. The genotypes of *VRTN* mutation of all pigs were determined using electrophoresis, in which a 411 bp fragment represented the mutant allele (*ins*) and a 120 bp represented the wild-type allele (*del*).

### Population structure and LD estimation

Principal component analysis (PCA) and LD analysis were performed using the SNPs that met the QC standards to investigate the population structure of the two Duroc pig populations. PCA was performed with GCTA [48]. LD among SNPs were estimated as the squared correlation *(r*^2^) of alleles with a window size of 1000. The average LD decay distance (*r*^2^ = 0.2) across the whole genome of the American and Canadian Duroc pigs was calculated by PLINK v1.07 [25]. Moreover, Weir & Cockerham *Fst* analysis was performed with the filtered SNPs using VCFtools [49] to estimate genetic differentiation between the two populations.

### Single-locus GWAS and meta-analysis

In the present study, the GEMMA software [16] was used to implement MLM for single-locus GWAS of teat number. The association between *VRTN* mutation and phenotypic data was analyzed using the identical MLM model employed by GEMMA [16]. GEMMA calculated the genomic relatedness matrix (GRM) between individuals within each population to account for population structure. The first five principal components calculated by GCTA tool were embedded as covariates in the association analysis model to eliminate the confounding effect of population structure [50]. The model that tested the allelic effect of each SNP on teat number invoked by GEMMA was as follows:

$$ \mathrm{y}=\mathrm{W}\upalpha +\mathrm{X}\upbeta +\mathrm{u}+\upvarepsilon $$

where y is the vector of teat number in these two Duroc pig populations; W is the incidence matrix of covariates (fixed effects) including sex and the top five eigenvectors of PCA; α is a vector of the corresponding coefficients including the intercept; X is the vector of all marker genotypes; β represents the corresponding effect of marker size; u refers to an n × 1 vector of random effects, with u ~ MVNn(0, λ τ^− 1^K), and ε is an vector of errors, with ε ~ MVNn(0, τ^− 1^*In*). τ^−1^ is the variance of the residual errors; K is GRM and λ represents the ratio between the two variance components; and I is the identity matrix, and n refers to the number of pigs. Given that Bonferroni correction is a stringent criterion, FDR was used to determine the threshold *P* values of single-locus GWAS [51, 52]. In the present study, FDR was set as 0.01, and the threshold *P* value was defined as *P = FDR × N/M*, where *N* represents the number of SNP’s *P* value < 0.01 in the results of GWAS and *M* refers to the total number of qualified SNPs for each trait of the two populations. Haploview v4.2 software [53] was employed for haplotype block analysis for the evaluation of the LD pattern of significant SNPs in an LD block because many significant SNPs were concentrated in the adjacent genomic regions of the chromosomes. Conditional analysis was conducted by fitting the genotypes of peak SNP as covariate to the univariate linear mixed model of GEMMA [16] to evaluate the presence of additional independent signals at each locus.

The phenotypic variance explained by genome-wide SNPs (SNP-based heritability) and the proportion of phenotypic variance explained by each significant SNPs were estimated using the model described as follows in GCTA [48]:

$$ \mathrm{y}=\mathrm{X}\upbeta +\mathrm{g}+\upvarepsilon\ \mathrm{with} \operatorname {var}\left(\mathrm{y}\right)={\mathrm{A}}_{\mathrm{g}}{\upsigma}_{\mathrm{g}}^2+\mathrm{I}{\upsigma}_{\upvarepsilon}^{2.} $$

where y is the vector of teat number; β is the vector including fixed effects; X is an incidence matrix for β; g is the vector of the aggregate effects of all the qualified 50 K SNPs for the pigs within one population; Ι is the identity matrix; A_g_ is the genomic relatedness matrix estimated by these SNPs; ${\upsigma}_{\mathrm{g}}^2$ is the additive genetic variance captured by either the genome-wide SNPs or the selected SNPs, and ${\upsigma}_{\upvarepsilon}^2$ is the residual variance. The phenotypic variance explained by the SNPs and *VRTN* mutation can be estimated using the model simply described as $\operatorname{var}\left({\mathrm{g}}_{\mathrm{snp}}\right)={\upsigma}_{\mathrm{g}}^2/{\upsigma}_{\mathrm{p}}^2$, where ${\upsigma}_{\mathrm{p}}^2$ (total phenotypic variance) is the sum of ${\upsigma}_{\mathrm{g}}^2$ and ${\upsigma}_{\upvarepsilon}^2$.

A meta-analysis of GWAS was performed on the two Duroc pig populations with METAL using a Z-score approach [54, 55]. In this study, the meta-analysis combined the effects and *P* values of 34,681 SNPs in the results of single-locus GWAS that were common to American and Canadian Duroc pigs.

### Multi-locus GWAS

Multi-locus GWAS was performed with four models, including mrMLM [18], FASTmrMLM [19], FASTmrEMMA [20] and ISIS EM-BLASSO [21]. All four multi-locus models were implemented in the R package “mrMLM” [18] to detect SNPs associated with teat number in two Duroc pig populations. Q (population genetic structure) matrix was the same as that used in single-locus GWAS and K (genomic relatedness matrix) matrix was calculated using R package “mrMLM” [18]. All SNPs were treated as random effects in the first stage of these four methods, in which the main purpose is to select all potentially relevant SNPs [23]. In the second stage, the selected SNPs were fitted into the multi-locus models and the markers with largest effects that surpassed the threshold of LOD values were regarded as promising trait-associated SNPs. The critical *P* value parameters were set at default values in the first step. The critical threshold of LOD score was set to 3 for SNPs at final stage [18].

### Identification of candidate genes and functional enrichment analysis

Based on the LD decay distances of the American and Canadian Duroc populations, the candidate genomic regions were determined to 540 kb and 800 kb on either side of the significant SNPs, respectively. Then, the genes within these regions were mined on Ensembl Sscrofa 11.1 (http://ensemble.org/Sus_scrofa/Info/Index) database to conduct gene enrichment analysis. Kyoto Encyclopedia of Genes and Genomes (KEGG) and Gene Ontology (GO) analyses were performed with all candidate genes using KOBAS 3.0 [56]. The enriched terms with the criteria of *P* < 0.05 were selected to further explore the genes involved in pathways and biological processes [52, 57].

## Supplementary information

**Additional file 1: Table S1.** Distributions of SNPs after QC and the average SNPs on each chromosome of American Duroc pigs.

**Additional file 2: Table S2.** Distributions of SNPs after QC and the average SNPs on each chromosome of Canadian Duroc pigs.

**Additional file 3: Figure S1.** SNP density and distribution across the genome. **a** SNP density on each chromosome of American origin population. **b** SNP density on each chromosome of Canadian origin population. The number of SNPs per kb in the consensus data set is shown as color index.

**Additional file 4: Figure S2.** Quantile-quantile (Q-Q) plots of single-locus GWAS for teat number in American origin **(a)** and Canadian origin **(b)** Duroc pig populations, respectively. Q-Q plots show the observed versus expected negative log 10 *P*-values.

**Additional file 5: Table S3.** The SNPs that surpassed the threshold *P-value* and were identified in the two Duroc pig populations by meta-analysis of GWAS.

**Additional file 6: Figure S3.** Manhattan plot of meta-analysis of GWAS for teat number in American and Canadian Duroc pigs. The dashed line indicates the FDR (0.01) thresholds for teat number (*P* = 1.18E-04).

**Additional file 7: Table S4.** Comparative mapping of tag SNPs with previous QTLs reported in the pig QTL database (as of March 24, 2020) and previous GWAS results.

**Additional file 8: Table S5.** Significant SNPs and genes associated with teat number in American Duroc pigs.

**Additional file 9: Table S6.** Significant SNPs and genes associated with teat number in Canadian Duroc pigs.

**Additional file 10: Table S7.** Significant KEGG pathways and GO terms associated with teat number. (*P* < 0.05).

## Data Availability

The datasets of genotypes analyzed during the current study are available on figshare (10.6084/m9.figshare.8019551.v1). The phenotypic data is not publicly available since the populations are consisted of the nucleus herd of Wens Foodstuff Group Co., Ltd., but are available from the corresponding author on reasonable request.

## Abbreviations

SSC: *Sus scrofa* chromosome
GWAS: Genome-wide association studiy
QTL: Quantitative trait locus
SNP: Single nucleotide polymorphism
LD: Linkage disequilibrium
MLM: Mixed linear model
mrMLM: Multiple-locus methods multi-locus random-SNP-effect mixed linear model
FASTmrMLM: Fast multiple-locus methods multi-locus random-SNP-effect mixed linear model
FASTmrEMMA: Fast multi-locus random-SNP-effect efficient mixed model association
ISIS EM-BLASSO: Integrative sure independence screening expectation maximization Bayesian least absolute shrinkage and selection operator model
