# Supplementary material for: Genome-wide association analyses identify known and novel loci for teat number in Duroc pigs using single-locus and multi-locus models
Source: BMC Genomics. 2020 May 7;21:344. doi: 10.1186/s12864-020-6742-6 (PMC7204245; doi:10.1186/s12864-020-6742-6)
Supplement: Supplementary file 1 — Additional file 1: Table S1. Distributions of SNPs after QC and the average SNPs on each chromosome of American Duroc pigs. [file 12864_2020_6742_MOESM1_ESM.docx]

**Table S1** Distributions of SNPs after QC and the average SNPs on each chromosome of American Duroc pigs

| SSC | SNP no. | chr. size (Mb)^a^ | SNP density (SNP/Mb) |
| --- | --- | --- | --- |
| 1 | 3531 | 274.33 | 12.87 |
| 2 | 2675 | 151.94 | 17.61 |
| 3 | 2389 | 132.85 | 17.98 |
| 4 | 2500 | 130.91 | 19.10 |
| 5 | 1884 | 104.53 | 18.02 |
| 6 | 2713 | 170.84 | 15.88 |
| 7 | 2322 | 121.84 | 19.06 |
| 8 | 2476 | 138.97 | 17.82 |
| 9 | 2516 | 139.51 | 18.03 |
| 10 | 1276 | 69.36 | 18.40 |
| 11 | 1635 | 79.17 | 20.65 |
| 12 | 1103 | 61.60 | 17.91 |
| 13 | 2944 | 208.33 | 14.13 |
| 14 | 2666 | 141.76 | 18.81 |
| 15 | 2472 | 140.41 | 17.61 |
| 16 | 1509 | 79.94 | 18.88 |
| 17 | 1178 | 63.49 | 18.55 |
| 18 | 1084 | 55.98 | 19.36 |

^a^The physical size is based on *Sus scrofa* Build 11.1 (http://www.ensembl.org/Sus_scrofa/Info/Index)
