# Supplementary material for: Genome-wide association analyses identify known and novel loci for teat number in Duroc pigs using single-locus and multi-locus models
Source: BMC Genomics. 2020 May 7;21:344. doi: 10.1186/s12864-020-6742-6 (PMC7204245; doi:10.1186/s12864-020-6742-6)
Supplement: Supplementary file 2 — Additional file 2: Table S2. Distributions of SNPs after QC and the average SNPs on each chromosome of Canadian Duroc pigs. [file 12864_2020_6742_MOESM2_ESM.docx]

**Table S2** Distributions of SNPs after QC and the average SNPs on each chromosome of Canadian Duroc pigs

| SSC | SNP no. | chr. Size (Mb)^a^ | SNP density (SNP/Mb) |
| --- | --- | --- | --- |
| 1 | 3250 | 274.33 | 11.85 |
| 2 | 2540 | 151.94 | 16.72 |
| 3 | 2104 | 132.85 | 15.84 |
| 4 | 2319 | 130.91 | 17.71 |
| 5 | 1745 | 104.53 | 16.69 |
| 6 | 2246 | 170.84 | 13.15 |
| 7 | 2140 | 121.84 | 17.56 |
| 8 | 2424 | 138.97 | 17.44 |
| 9 | 2333 | 139.51 | 16.72 |
| 10 | 1193 | 69.36 | 17.20 |
| 11 | 1494 | 79.17 | 18.87 |
| 12 | 1013 | 61.60 | 16.44 |
| 13 | 2813 | 208.33 | 13.50 |
| 14 | 2520 | 141.76 | 17.78 |
| 15 | 2240 | 140.41 | 15.95 |
| 16 | 1465 | 79.94 | 18.33 |
| 17 | 1119 | 63.49 | 17.62 |
| 18 | 975 | 55.98 | 17.42 |

^a^The physical size is based on *Sus scrofa* Build 11.1 (http://www.ensembl.org/Sus_scrofa/Info/Index)
