# Supplementary figures and images for: Genome-wide association analyses identify known and novel loci for teat number in Duroc pigs using single-locus and multi-locus models
Source: BMC Genomics. 2020 May 7;21:344. doi: 10.1186/s12864-020-6742-6 (PMC7204245; doi:10.1186/s12864-020-6742-6)

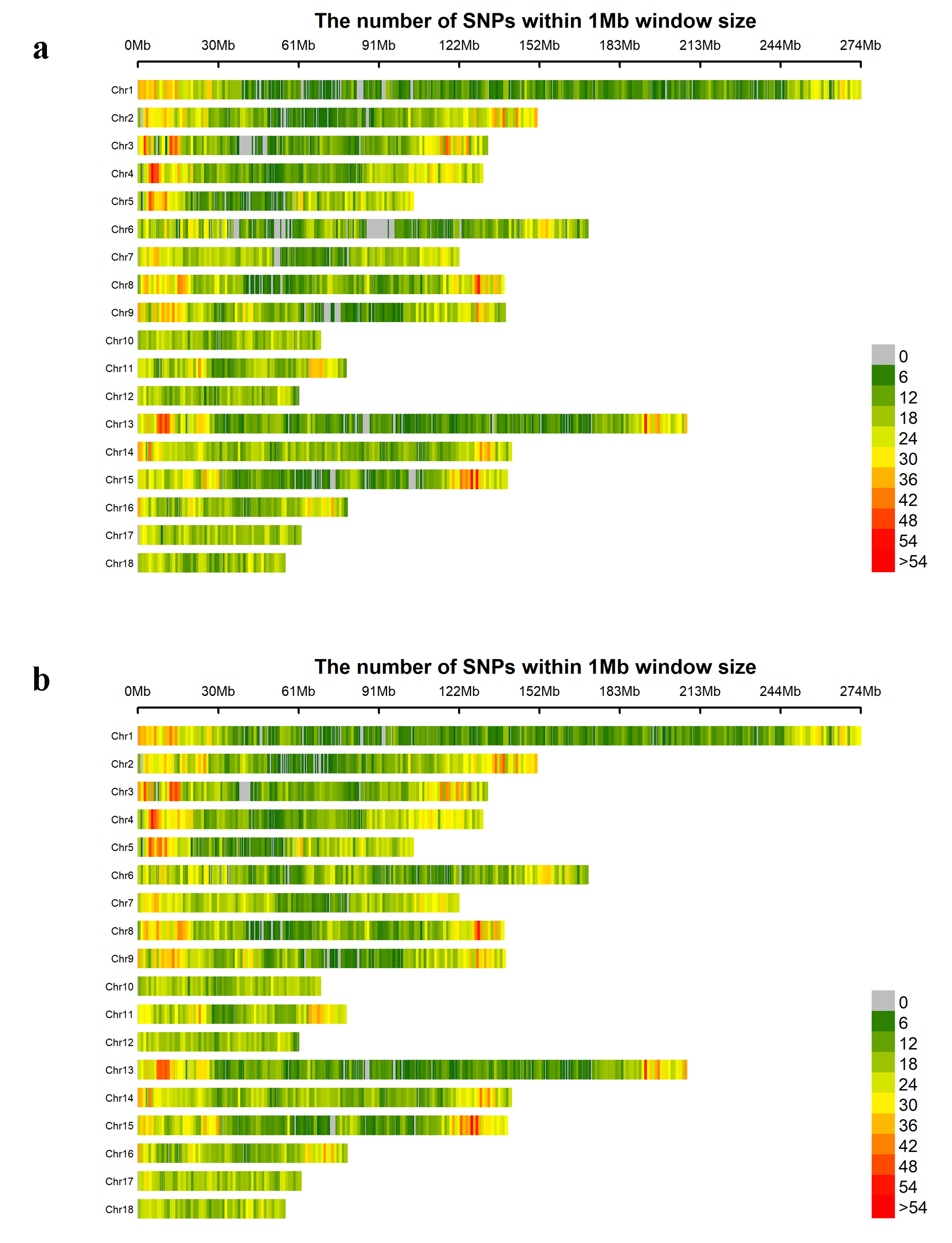

Supplement: Supplementary file 3 — Additional file 3: Figure S1. SNP density and distribution across the genome. a SNP density on each chromosome of American origin population. b SNP density on each chromosome of Canadian origin population. The number of SNPs per kb in the consensus data set is shown as color index. [file 12864_2020_6742_MOESM3_ESM.jpg]

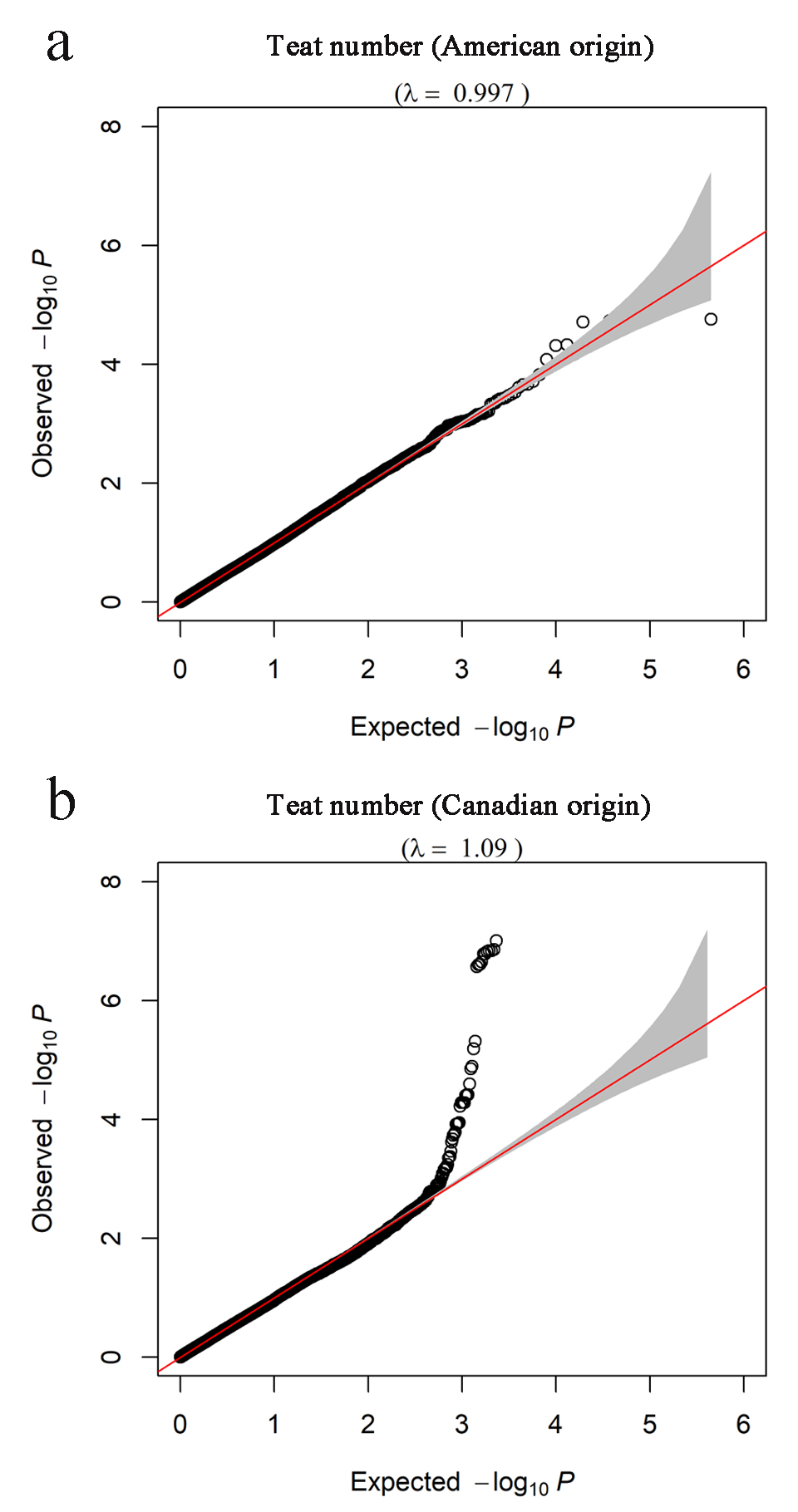

Supplement: Supplementary file 4 — Additional file 4: Figure S2. Quantile-quantile (Q-Q) plots of single-locus GWAS for teat number in American origin (a) and Canadian origin (b) Duroc pig populations, respectively. Q-Q plots show the observed versus expected negative log 10 P-values. [file 12864_2020_6742_MOESM4_ESM.jpg]

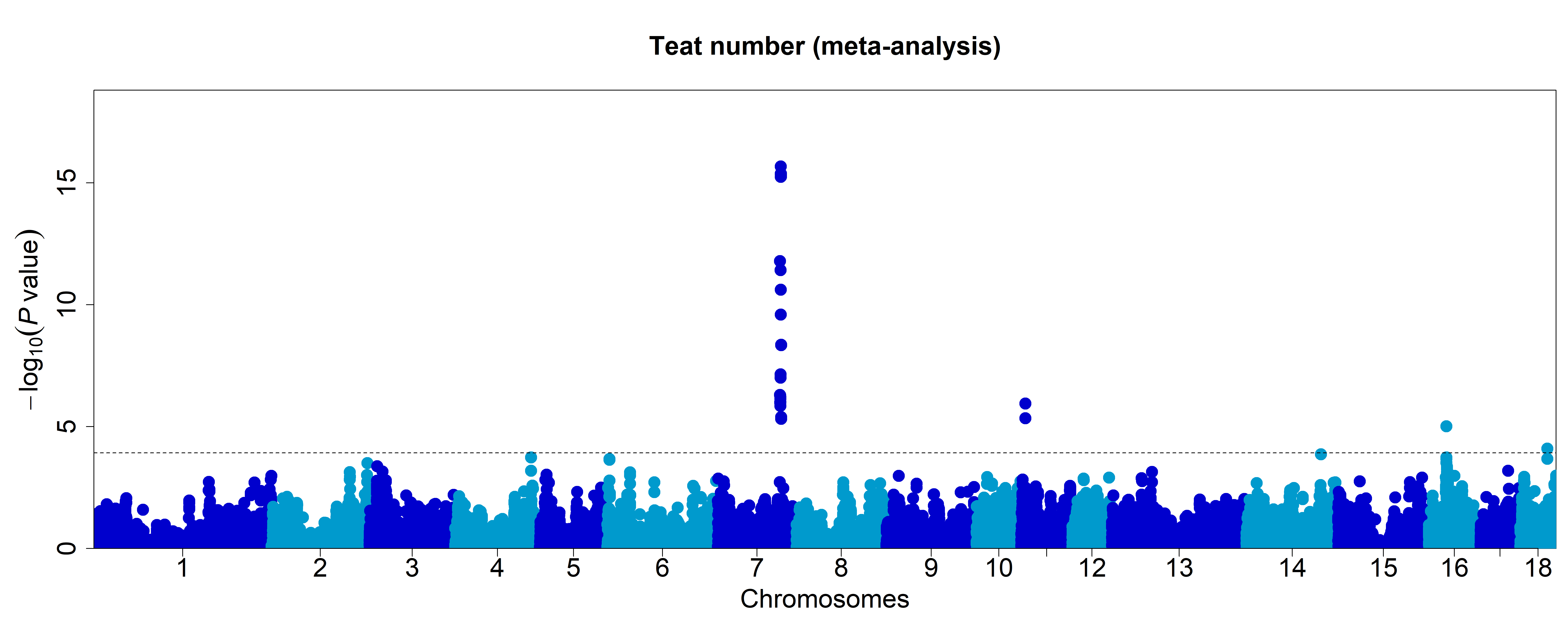

Supplement: Supplementary file 6 — Additional file 6: Figure S3. Manhattan plot of meta-analysis of GWAS for teat number in American and Canadian Duroc pigs. The dashed line indicates the FDR (0.01) thresholds for teat number (P = 1.18E-04). [file 12864_2020_6742_MOESM6_ESM.png]
