# Supplementary material for: Genome-wide association analyses identify known and novel loci for teat number in Duroc pigs using single-locus and multi-locus models
Source: BMC Genomics. 2020 May 7;21:344. doi: 10.1186/s12864-020-6742-6 (PMC7204245; doi:10.1186/s12864-020-6742-6)
Supplement: Supplementary file 7 — Additional file 7: Table S4. Comparative mapping of tag SNPs with previous QTLs reported in the pig QTL database (as of March 24, 2020) and previous GWAS results. [file 12864_2020_6742_MOESM7_ESM.docx]

**Table S4** Comparative mapping of tag SNPs with previous QTLs reported in the pig QTL database (as of March 24, 2020) and previous GWAS results

| SSC^a^ | SNP ID^b^ | Location (bp)^c^ | QTL location range^d^ | QTL ID^e^ | Related QTL^f^ | Trait^g^ |
| --- | --- | --- | --- | --- | --- | --- |
| 1 | **rs81296766** | 26423644 | 1:10067182-292041505 | 21208 | Backfat at last rib | TN |
| 1 | rs81353367 | 32198014 | NA | NA | NA | TN |
| 1 | rs81354014 | 49632560 | 1:49026306-49988544 | 126635 | Teat number | TN |
| 1 | rs80808645 | 50037586 | 1:45939845-292033322 | 5223 | Teat number | TN |
| 1 | rs80855587 | 166253306 | 1:45939845-292033323 | 5223 | Teat number | TN |
| 1 | rs80805477 | 248169556 | 1:45939845-292033324 | 5223 | Teat number | TN |
| 1 | rs81315010 | 251816790 | 1:45939845-292033325 | 5223 | Teat number | TN |
| 1 | rs333890665 | 271354424 | 1:45939845-292033325 | 5223 | Teat number | TN |
| 1 | rs321500205 | 271382273 | 1:45939845-292033326 | 5223 | Teat number | TN |
| 2 | rs81356579 | 27110325 | 2:13366532-44483418 | 909 | Teat number | TN |
| 2 | **rs330333016** | 77481598 | 2:609757-85009446 | 16875 | Backfat at last rib | TN |
| 2 | **rs81363870** | 121140488 | 2:13366532-154953107 | 5685 | Backfat at last rib | TN |
| 2 | **rs338630193** | 127747767 | 2:13366532-154953107 | 5685 | Backfat at last rib | TN |
| 3 | rs344649466 | 5715228 | 3:1456046-130209174 | 5224 | Teat number | TN |
| 3 | rs81314408 | 20394893 | 3:1456046-130209174 | 5224 | Teat number | TN |
| 3 | rs81338014 | 32426287 | 3:1456046-130209174 | 5224 | Teat number | TN |
| 4 | **rs318980859** | 117518657 | 4:102054630-140378854 | 7473 | Nonfunctional nipples | TN |
| 5 | rs341491167 | 10879898 | 5:844337-34660429 | 2927 | Teat number | TN |
| 5 | rs81384813 | 66721316 | 5:11496289-77598894 | 24288 | Teat number | TN |
| 5 | rs81384838 | 66734388 | 5:11496289-77598894 | 24288 | Teat number | TN |
| 5 | rs328599079 | 75707707 | 5:11496289-77598894 | 24288 | Teat number | TN |
| 6 | rs81395407 | 35040992 | 6:2352681-72944202 | 24289 | Teat number | TN |
| 6 | rs333592328 | 49265869 | 6:2352681-72944202 | 24289 | Teat number | TN |
| 6 | rs81389632 | 89786916 | 6:19536155-157765593 | 5226 | Teat number | TN |
| 6 | rs81391820 | 134798234 | 6:19536155-157765593 | 5226 | Teat number | TN |
| 6 | **rs324552394** | 164807745 | NA | NA | NA | TN |
| 6 | **rs705289935** | 168268278 | NA | NA | NA | TN |
| 7 | **rs80864749** | 7655911 | 7:4252710-11625414 | 3860 | Backfat at tenth rib | TN |
| 7 | **rs330783620** | 9154293 | 7:4252710-11625414 | 3860 | Backfat at tenth rib | TN |
| 7 | **rs80964371** | 92809231 | 7:4252710-11625414 | 3860 | Backfat at tenth rib | TN |
| 7 | rs80888936 | 96128654 | 7:88946228-107293999 | 8805 | Teat number | TN |
| 7 | rs324614194 | 96278617 | 7:88946228-107293999 | 8805 | Teat number | TN |
| 7 | rs331807204 | 96632217 | 7:88946228-107293999 | 8805 | Teat number | TN |
| 7 | rs81265875 | 96660861 | 7:88946228-107293999 | 8805 | Teat number | TN |
| 7 | rs329434246 | 96694364 | 7:88946228-107293999 | 8805 | Teat number | TN |
| 7 | rs81396029 | 96727497 | 7:88946228-107293999 | 8805 | Teat number | TN |
| 7 | rs81295281 | 96731838 | 7:88946228-107293999 | 8805 | Teat number | TN |
| 7 | rs81396040 | 96743525 | 7:88946228-107293999 | 8805 | Teat number | TN |
| 7 | rs81227580 | 96786714 | 7:88946228-107293999 | 8805 | Teat number | TN |
| 7 | rs81396043 | 96806775 | 7:88946228-107293999 | 8805 | Teat number | TN |
| 7 | rs342685919 | 97048514 | 7:88946228-107293999 | 8805 | Teat number | TN |
| 7 | rs80843834 | 97109772 | 7:88946228-107293999 | 8805 | Teat number | TN |
| 7 | rs80805264 | 97126583 | 7:88946228-107293999 | 8805 | Teat number | TN |
| 7 | rs327357811 | 97347282 | 7:88946228-107293999 | 8805 | Teat number | TN |
| 7 | rs319296259 | 97394296 | 7:88946228-107293999 | 8805 | Teat number | TN |
| 7 | rs346287309 | 97427849 | 7:88946228-107293999 | 8805 | Teat number | TN |
| 7 | rs692640845 | 97568284 | 7:88946228-107293999 | 8805 | Teat number | TN |
| 7 | rs1113960993 | 97575068 | 7:88946228-107293999 | 8805 | Teat number | TN |
| 7 | rs330032123 | 97584287 | 7:88946228-107293999 | 8805 | Teat number | TN |
| 7 | VRTN_mutation | 97615880 | 7:88946228-107293999 | 8805 | Teat number | TN |
| 7 | rs343248943 | 97617907 | 7:88946228-107293999 | 8805 | Teat number | TN |
| 7 | rs80894106 | 97652632 | 7:88946228-107293999 | 8805 | Teat number | TN |
| 7 | rs81238639 | 97946666 | 7:88946228-107293999 | 8805 | Teat number | TN |
| 7 | rs80864705 | 97954258 | 7:88946228-107293999 | 8805 | Teat number | TN |
| 7 | rs80929215 | 97973860 | 7:88946228-107293999 | 8805 | Teat number | TN |
| 7 | rs80813473 | 98066911 | 7:88946228-107293999 | 8805 | Teat number | TN |
| 7 | rs80836267 | 98089286 | 7:88946228-107293999 | 8805 | Teat number | TN |
| 7 | rs80865802 | 102479725 | 7:88946228-107293999 | 8805 | Teat number | TN |
| 7 | rs338075156 | 102513443 | 7:88946228-107293999 | 8805 | Teat number | TN |
| 7 | rs80975884 | 102552105 | 7:88946228-107293999 | 8805 | Teat number | TN |
| 7 | rs80822795 | 102658822 | 7:88946228-107293999 | 8805 | Teat number | TN |
| 7 | rs80795811 | 103109678 | 7:88946228-107293999 | 8805 | Teat number | TN |
| 7 | NA | 103132435 | 7:88946228-107293999 | 8805 | Teat number | TN |
| 7 | rs80847916 | 103151323 | 7:88946228-107293999 | 8805 | Teat number | TN |
| 7 | NA | 103164950 | 7:88946228-107293999 | 8805 | Teat number | TN |
| 8 | rs81401285 | 72626638 | 8:38873367-90653103 | 4253 | Teat number | TN |
| 8 | rs343488415 | 73599016 | 8:38873367-90653103 | 4253 | Teat number | TN |
| 8 | **rs81335362** | 136866026 | 8:108610930-139007531 | 17782 | Backfat at last rib | TN |
| 9 | **rs81420227** | 14459452 | 9:11066889-23144816 | 5711 | Body weight (weaning) | TN |
| 10 | rs334392548 | 16387485 | 10:10524710-44992400 | 5259 | Teat number | TN |
| 11 | rs80803790 | 6291044 | 11:1111096-68683528 | 5260 | Teat number | TN |
| 11 | rs80914601 | 6324834 | 11:1111096-68683528 | 5260 | Teat number | TN |
| 11 | rs343377111 | 15473025 | 11:1111096-68683528 | 5260 | Teat number | TN |
| 11 | rs81305437 | 25782658 | 11:1111096-68683528 | 5260 | Teat number | TN |
| 11 | rs80809451 | 34924123 | 11:1111096-68683528 | 5260 | Teat number | TN |
| 11 | **rs80930723** | 70370312 | 11:62760744-81069519 | 3965 | Number of ribs | TN |
| 12 | rs81440983 | 17695233 | 12:10361588-63588571 | 5261 | Teat number | TN |
| 13 | **rs335055280** | 244235 | 13:88939-5747388 | 25 | Backfat at tenth rib | TN |
| 13 | **rs345752157** | 198613309 | 13:194995520-206658205 | 21404 | Backfat at last rib | TN |
| 14 | **rs345307243** | 24753992 | 14:6898350-27531879 | 16841 | Backfat at last rib | TN |
| 14 | **rs80848162** | 26467369 | 14:6898350-27531879 | 16841 | Backfat at last rib | TN |
| 14 | **rs81450840** | 57407462 | 14:47658364-58439670 | 121910 | Backfat between 3rd and 4th last ribs | TN |
| 14 | **rs80890762** | 69437518 | 14:27531879-132170772 | 16873 | Carcass weight (hot) | TN |
| 14 | **rs321772507** | 115176455 | 14:27531879-132170772 | 16873 | Carcass weight (hot) | TN |
| 14 | **rs327004523** | 133536115 | 14:7424787-152596119 | 5233 | Body weight (birth) | TN |
| 14 | **rs80823799** | 136408216 | 14:7424787-152596119 | 5233 | Body weight (birth) | TN |
| 14 | **rs80794466** | 137506343 | 14:7424787-152596119 | 5233 | Body weight (birth) | TN |
| 15 | **rs80957887** | 111327896 | 15:46030500-149797711 | 3975 | Backfat at first rib | TN |
| 15 | **rs333698977** | 129904530 | 15:46030500-149797711 | 3975 | Backfat at first rib | TN |
| 16 | rs322985099 | 6124952 | 16:342954-79624164 | 5228 | Teat number | TN |
| 16 | rs81316660 | 27084056 | 16:342954-79624164 | 5228 | Teat number | TN |
| 16 | rs81461904 | 69106054 | 16:342954-79624164 | 5228 | Teat number | TN |
| 17 | **rs80843610** | 8468654 | 17:435637-19243638 | 12538 | Backfat at last rib | TN |
| 17 | rs319134655 | 44791974 | 17:16015836-69701581 | 5229 | Teat number | TN |
| 18 | **rs321942793** | 6398431 | 18:5665843-27641334 | 3301 | Backfat at last rib | TN |
| 18 | **rs81469480** | 42123454 | NA | NA | NA | TN |
| 18 | rs81471144 | 51455200 | 18:50123715-51644236 | 37473 | Teat number | TN |

^a^ Sus scrofa chromosome ^b^ The bold data indicate newly discovered SNPs associated with teat number ^c^ SNP position in Ensembl ^d^ Location range of the mapped QTL in the QTL database ^e^ Identity of QTL in the pig QTL database or published literature ^f^ The name of mapped QTL in the database (<https://www.animalgenome.org/cgi-bin/QTLdb/SS/index>) ^g^ Teat number
